# Immune checkpoint inhibitor-associated diabetic ketoacidosis induced by cadonilimab: a case report and literature review

**DOI:** 10.3389/fendo.2026.1819809

**Published:** 2026-04-14

**Authors:** Yuchen Jiang, Yu Cui, Dan Su, Hao Yang, Xi Chen, Xiaodan Qian, Lidan Tang, Shan Xu

**Affiliations:** 1 Department of Medicine Storehouse, The Second People’s Hospital of Changzhou, the Third Affiliated Hospital of Nanjing Medical University, Changzhou, Jiangsu, China; 2 Department of Pharmacy, The Second People’s Hospital of Changzhou, the Third Affiliated Hospital of Nanjing Medical University, Changzhou, Jiangsu, China; 3 College of Pharmacy, Dalian Medical University, Dalian, Liaoning, China; 4 Department of Pharmacy, Xuzhou First People’s Hospital, Xuzhou, Jiangsu, China

**Keywords:** cadonilimab, case report, diabetic ketoacidosis, immune checkpoint inhibitor induced type 1 diabetes mellitus (ICI-T1DM), literature review

## Abstract

Immune checkpoint inhibitors (ICIs) significantly improve prognosis and survival outcomes in cancer patients by enhancing immune function, thereby providing new therapeutic hope for cancer patients. However, with the widespread clinical application of ICIs, an increasing number of immune-related adverse events (irAEs) have been reported. Immune checkpoint inhibitor-induced type 1diabetes mellitus (ICI-T1DM) is a rare but potentially life-threatening irAE, usually presenting as acute onset and easily progressing to diabetic ketoacidosis (DKA) or hyperglycemic hyperosmolar state (HHS), which poses a serious threat to patients’ safety. This study reports a case of DKA in an 81-year-old female patient diagnosed with cervical squamous cell carcinoma without history of diabetes mellitus, which developed after multiple cycles of Cadonilimab. The patient’s blood glucose levels were effectively controlled via insulin therapy and fluid resuscitation, and a definitive diagnosis of ICI-T1DM was confirmed. Taking this case as a starting point, this article reviews the epidemiology, clinical characteristics, pathogenesis, and clinical management strategies of ICI-T1DM, aiming to enhance clinicians’ awareness of ICI-T1DM, especially the endocrine toxicity of dual-target ICIs such as cadonilimab, and provide practical reference for ensuring the safety of ICI therapy in cancer patients.

## 1 Introduction

Cadonilimab (AK104), independently developed by Kangfang Biotechnology (China), is the world’s first clinically approved bispecific antibody for tumor immunotherapy targeting both programmed death-1 (PD-1) and cytotoxic T lymphocyte-associated antigen-4 (CTLA-4) (1). Its anti-tumor mechanism lies in dual regulation of immune checkpoints: blocking the PD-1 pathway reverses T-cell exhaustion in the tumor microenvironment, while inhibiting the CTLA-4 pathway enhances early T-cell activation in lymph nodes, thereby achieving a synergistic anti-tumor effect (2). In June 2022, China’s National Medical Products Administration (NMPA) approved cadonilimab for the second-line treatment of recurrent or metastatic cervical cancer. A Phase III clinical trial (NCT04982237) is now evaluating its efficacy as a first-line therapy in combination with standard chemotherapy for this condition (3).

Notably, PD-1 and PD-L1 are expressed not only on immune cells but also in various normal tissues, including pancreatic β-cells, hematopoietic cells, and thyroid follicular cells (2). Consequently, by activating an anti-tumor immune response, ICIs can disrupt immune tolerance in these tissues and trigger a spectrum of irAEs. Endocrine system-related irAEs, such as thyroiditis, hypophysitis, and adrenal insufficiency, are among the more frequently observed (2). Although ICI-T1DM represents a rarer subtype of endocrine irAE, it carries a high risk of severe complications like DKA and a poor prognosis if not promptly managed.

To date, no cases of cadonilimab-induced diabetic ketoacidosis (DKA) have been reported in clinical trials or real-world evidence, and data on its endocrine toxicity remain limited. We report a case of DKA following cadonilimab monotherapy in a patient with cervical cancer. By integrating a review of the literature, we analyze the clinical features and management of immune checkpoint inhibitor-induced type 1 diabetes mellitus (ICI-T1DM). This case supplements the safety profile of dual-target immune checkpoint inhibitors and offers guidance for clinical practice.

## 2 Case report

### 2.1 Patient baseline characteristics

An 81-year-old female patient, with a height of 155 cm and weight of 52 kg (BMI 21.6 kg/m²), was diagnosed with cervical squamous cell carcinoma (cT2bNXM0, Stage IIB) in March 2023. The patient had no history of chronic diseases, including hypertension, diabetes mellitus, hepatitis, or tuberculosis, and reported no food or drug allergies. Her medical history included two prior episodes of cerebral infarction, which left no residual neurological deficits, and cataract surgery performed ten years ago with good postoperative recovery. She was not taking any regular medications prior to admission. The relatives are in good health, with no history of the same disease, infectious diseases, and genetic disorders such as diabetes, cancer, hypertension, and etc.

### 2.2 Treatment course before admission

The patient initiated radiotherapy for cervical cancer in April 2023 but discontinued it after seven sessions due to severe lower abdominal pain, increased vaginal bleeding, fatigue, and anorexia. From July to August 2023, she received traditional Chinese medicine for symptom relief, though the specific components and dosages were not documented. Between October 11 and November 21, 2023, she completed three cycles of combined therapy with cadonilimab (250 mg via intravenous infusion every three weeks) and anlotinib (12 mg orally once daily, administered for two weeks followed by a one-week break per three-week cycle). This combined treatment significantly alleviated her vaginal bleeding and abdominal pain, and a subsequent CT scan indicated a partial response (PR) of the tumor. Anlotinib was discontinued following a new diagnosis of cerebral infarction on December 25, 2023, after which cadonilimab monotherapy was continued until January18 2024. No significant adverse reactions were noted until one week prior to admission, when she developed recurrent abdominal pain without accompanying vaginal bleeding, nausea, vomiting, or fever.

### 2.3 Admission manifestations and DKA diagnosis

The patient was admitted on January 17, 2024, with an initial diagnosis of cervical malignant tumor (cT2bNXM0, Stage IIB) and a personal history of cerebral infarction. On admission, her vital signs were temperature 36.7 °C, pulse 78 beats/min, respiratory rate 14 breaths/min, and blood pressure 118/76 mmHg. Physical examination of the heart, lungs, and abdomen revealed no obvious abnormalities, and the neurological examination showed no positive signs.

On January 17, she developed frequent nausea and vomiting, accompanied by small amounts of hematemesis. Treatment with metoclopramide, Crotalus tigrinus snake venom coagulant, and carbazochrome sodium sulfonate did not significantly improve her symptoms. Emergency laboratory tests on January 18 revealed severe hyperglycemia, with a blood glucose level exceeding 34.69 mmol/L. A subcutaneous injection of 10 U of insulin was administered immediately, but her blood glucose remained uncontrolled. Further examinations confirmed the diagnosis of diabetic ketoacidosis (DKA) (4, 5) with concurrent upper gastrointestinal bleeding (**Table 1**).

**Table 1:** Key laboratory results recorded during the patient’s hospitalization.

Date	Examination items	Laboratory result	Reference range for hospital laboratories
Jan 18, 2024	Glucose (mmol/L)	>34.69	4.1~5.9
	Urea (mmol/L)	11.0	2.5~6.1
	Creatinine (μmol/L)	129.9	46~92
	Blood pH	7.2	7.35~7.45
	Carbon dioxide partial pressure(KPa)	2.5	4.65~5.98
	Actual bicarbonate (mmol/L)	7.4	21~28
	Total carbon dioxide (mmol/L)	8.0	24~32
	Base excess (mmol/L)	-18.6	-3~3
	Serum C-peptide (pmol/L)	12.3	370~1470
	Urinary ketones	1+	negative
	Insulin(pmol/L)	32.5	17.8~173
	Anti-glutamic acid decarboxylase antibody(IU/mL)	negative	0~10
	Anti-insulin antibody(RU/mL)	negative	0~20
Jan 19, 2024	Blood pH	7.34	7.35~7.45
	Glucose (mmol/L)	16.44	4.1~5.9
	Base excess (mmol/L)	-5.2	-3~3
Jan 22, 2024	Glucose (mmol/L)	5.8-15.6	4.1~5.9
	Serum cortisol (μg/dL)	12.41	8.7~22.4
	Adrenocorticotropic hormone 8 am(pmol/L)	3.10	1.6~13.9
	HbA1c (%)	6.8	4~6
	Urinary ketones	2+	negative
	Blood pH	7.5	7.35~7.45
	Actual bicarbonate (mmol/L)	28.9	21~28
	Alkaline surplus (mmol/L)	5.5	-3~3

### 2.4 Treatment and outcome of DKA

Following consultation with endocrinology and clinical pharmacy, a treatment plan was implemented: a) Cadonilimab was discontinued, suspending ICI therapy due to suspected ICI-DM. b) For glycemic control, continuous subcutaneous insulin infusion (CSII) with insulin aspart was initiated at 0.1 U/kg/h, with titration guided by hourly blood glucose monitoring (values ranged from 8.5 to 28.1 mmol/L on the first day). c) Fluid and electrolyte replacement involved infusion of 0.9% normal saline (1000 mL within the first 2 hours) for volume repletion, with close monitoring and supplementation of serum potassium when levels fell below 3.5 mmol/L/d) Gastrointestinal bleeding was managed with a continuous somatostatin infusion until cessation, after which oral hydration was resumed.

**Figure 1:**
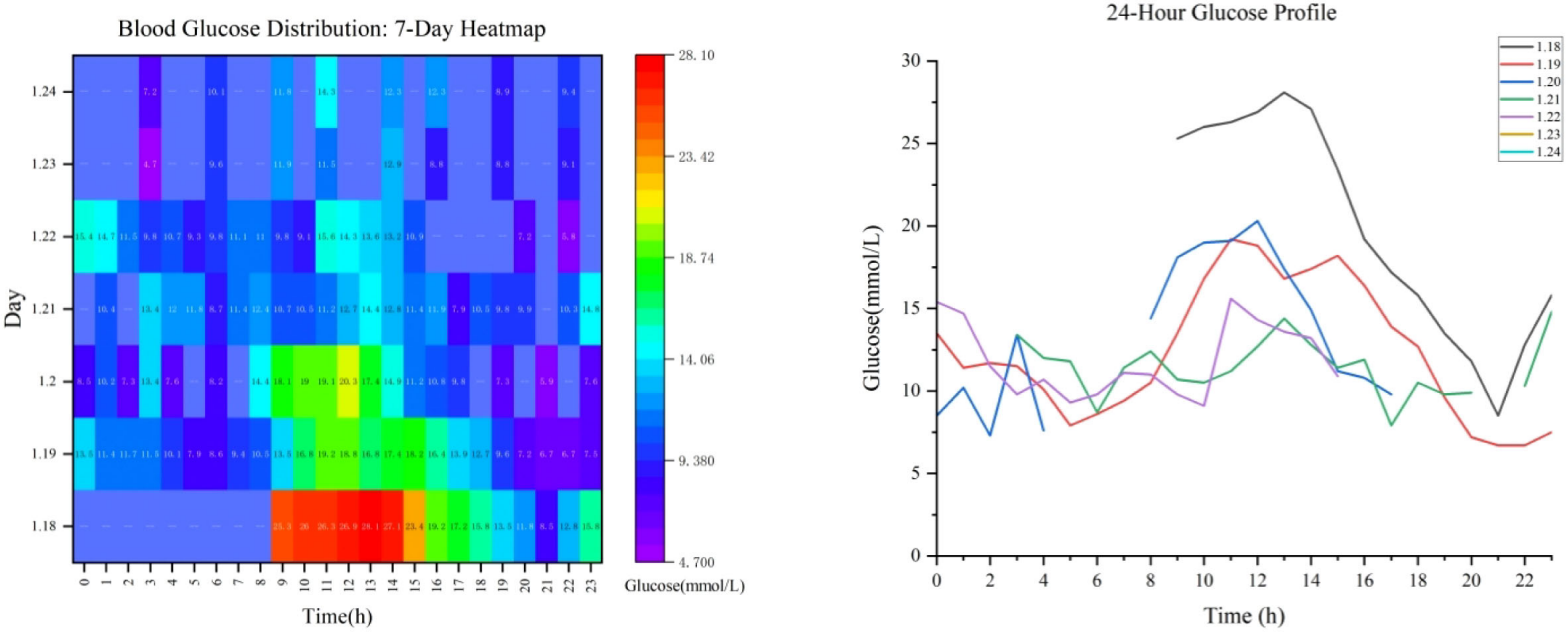
Dynamic changes in blood glucose levels during the patient’s hospitalization.

By January 19, the patient’s metabolic acidosis had significantly improved (blood pH 7.34, base excess -5.2 mmol/L), with resolution of nausea and vomiting and no further hematemesis; somatostatin was stopped and oral intake was gradually reintroduced. On January 22, the insulin regimen was transitioned from CSII to multiple daily injections (MDI), comprising 10 U of insulin glargine at bedtime and 6 U of insulin aspart 30 minutes before each meal, which maintained blood glucose between 5.8 and 15.6 mmol/L. The onset of Cadonilimab-induced type 1 diabetes was on January 18, 2024. Insulin therapy was maintained long-term and not discontinued, because ICI-DM results from irreversible pancreatic β-cell destruction and requires lifelong insulin replacement. By January 26, the patient exhibited no recurrence of abdominal pain, vomiting, or bleeding, and her oral intake had normalized, leading to discharge with diagnoses of cervical malignant tumor (cT2bNXM0, Stage IIB), personal history of cerebral infarction, diabetic ketoacidosis, and upper gastrointestinal bleeding. **Figure 1** illustrates the dynamic 24-hour blood glucose profile during the patient’s hospitalization.

## 3 Discussion

### 3.1 Causal association between DKA and cadonilimab

The patient had no prior personal and family history of diabetes, indicating that the current DKA episode was likely drug-induced. The causal association was assessed using established criteria for adverse drug reactions: a plausible temporal relationship existed between Cadonilimab administration and symptom onset, and the drug’s prescribing information lists diabetes as a potential adverse effect. The patient’s hyperglycemia responded to insulin therapy, and tumor progression were excluded as a probable cause. Re-challenge with the drug was not performed, precluding an evaluation of symptom recurrence. Although anlotinib was administered previously and may affect glucose metabolism and insulin sensitivity, and may have a synergistic effect with Cadonilimab to increase the risk of immune-mediated pancreatic β−cell damage and severe DKA. Based on this assessment, Cadonilimab is considered the primary and dominant cause of ICI-DM and DKA in this case, the combined effect of the two drugs cannot be completely excluded. Upon admission, the patient’s random blood glucose exceeded 34.69 mmol/L, which corresponds to a Grade 4 adverse reaction according to the NCCN (4) and CSCO guidelines for immune checkpoint inhibitor toxicity management (6).

### 3.2 Literature review on ICI-T1DM

Immune checkpoint inhibitors (ICI) have markedly improved the prognosis and survival of cancer patients by enhancing antitumor immune responses. Their expanding clinical use, however, has been accompanied by a rise in reported adverse events, among which endocrine toxicities are particularly frequent (1). These include thyroiditis, hypophysitis, diabetes mellitus, and primary adrenal insufficiency (2, 3). ICI-induced type 1 diabetes mellitus (ICI-T1DM) is a rare but potentially life-threatening adverse reaction that typically manifests with acute onset, which can progress to severe hyperglycemic hyperosmolar states or diabetic ketoacidosis (DKA). ICI-T1DM is typically irreversible, resulting from the permanent and severe destruction of pancreatic islet β cells. Corticosteroids are not recommended for treatment, as they are ineffective and may exacerbate hyperglycemia or increase the risk of DKA. Dual immune checkpoint blockade combination therapy causes more extensive and severe β-cell damage than monotherapy, which correlates with the severity and prognosis of the diabetes.

#### 3.2.1 Epidemiology

Wu et al. (5) defined ICI-T1DM by the concurrent presence of diabetes (blood glucose >11 mmol/L or HbA1c≥6.5%) and insulin deficiency (C-peptide <0.4 nmol/L or DKA), provided the patient was not taking an SGLT2 inhibitor. The Chinese Guidelines for the Prevention and Treatment of Type 2 Diabetes (2020 Edition) (7) state that DKA can be diagnosed with elevated blood ketones (≥23 mmol) or strongly positive urine glucose and ketones (++ or higher) alongside hyperglycemia (>13.9 mmol), when accompanied by a blood pH<7.3 and/or a bicarbonate level <18 mmol/L, irrespective of diabetes history.

ICI-T1DM is a rare adverse event. Zhen et al. reported a 1.01% incidence of new-onset or worsening diabetes (8), while multiple retrospective studies indicate an incidence ranging from approximately 0.8% to 1.9% (2, 9–13). ICI-T1DM occurs predominantly in males and typically manifests early during ICI treatment (14). More than 60% of patients present with DKA (15–20), whose initial symptoms include polyuria, polydipsia, and weight loss. Other common features are abdominal pain, nausea, vomiting, and hyperventilation, and some patients develop neurological symptoms such as altered mental status (ranging from somnolence to coma) or focal neurological signs (21). ICI-T1DM is frequently associated with pancreatic atrophy and pancreatitis (18); consequently, assessing pancreatic volume via CT may help prevent diabetic emergencies (22). Laboratory findings typically reveal a rapid, sustained, and severe decline in insulin and C-peptide levels alongside hyperglycemia, with some patients showing elevated lipase (18). Compared to traditional type 1 diabetes, ICI-T1DM is characterized by an older age of onset, more rapid progression, and lower rates of antibody positivity (5, 20). This case exhibited the classic HbA1c paradox characteristic of ICI-induced type 1 diabetes: the patient’s blood glucose was severely elevated to over 34.69 mmol/L, while HbA1c was only mildly increased to 6.8%. This distinct pattern indicates acute-onset diabetes resulting from rapid pancreatic β-cell destruction, not chronic hyperglycemia. Consequently, the HbA1c paradox provides a critical diagnostic clue for confirming ICI-T1DM and distinguishing it from type 2 diabetes mellitus.

#### 3.2.2 Pathogenesis and risk factors

The pathogenesis of ICI-T1DM remains incompletely understood. One proposed mechanism is that it represents a novel form of autoinflammatory β-cell failure (11). This onset may be linked to abnormal PD-1 ligand expression on pancreatic β cells, ICI-induced disruption of local pancreatic immune balance, and humoral or inflammatory responses within the pancreas (23). In this autoimmune process, activated T cells are central, with genetic and environmental factors contributing to the destructive cascade (19). Blocking the PD-1/PD-L1 interaction is thought to stimulate T-cell proliferation and activation, leading to β-cell destruction and diabetes (24). Furthermore, genetic analyses have identified variants in genes such as ORM1, PLG, and G6PC, which are associated with type 1 diabetes or pancreatic function, alongside missense mutations in NLRC5 (25). Although NLRC5 is also linked to T1DM, no germline NLRC5 mutations are recorded in public T1DM databases, suggesting it may not be a risk factor for T1DM and indicating that ICI-T1DM may involve a distinct genetic susceptibility (25).

The risk of ICI-T1DM varies with tumor type and the specific immune checkpoint inhibitor administered. A systematic review of 172 ICI-DM cases reported melanoma (43.6%; 75/172), lung cancer (30.2%; 52/172), renal cell carcinoma (5.8%; 10/172), breast cancer (3.5%; 6/172), gastrointestinal cancer (3.5%; 6/172), lymphoma (2.9%; 5/172), and hepatocellular carcinoma (1.2%; 2/172) as the most common malignancies (15). ICI-T1DM is most frequently observed with PD-1/PD-L1 monoclonal antibody therapy, with no cases yet reported for Cadonilimab. Zhao et al. found pembrolizumab was associated with an elevated risk of all endocrine disorders, including diabetes, with melanoma patients at higher risk for hypophysitis and diabetes (26). Chen et al. reported that combined CTLA-4 and PD-1/PD-L1 blockade carried a higher risk of ICI-T1DM compared to anti-PD-(L)1 monotherapy (*HR* = 1.62; 95% *CI* = 1.15-2.26) *(*10), a finding supported by other studies (27, 28). In addition to those that have not yet been reported or clearly defined, based on the literature and package insert, the immune checkpoint inhibitors (ICIs) with the lowest reported occurrence rate of ICI-T1DM are Durvalumab and Ipilimumab (incidence <0.1%), as detailed in **Table 2**.

**Table 2:** ICI approved in china and associated incidence of ICI-T1DM.

Category	Drug name (Generic)	Manufacturer	Incidence of ICI-T1DM
PD-1 inhibitor	Nivolumab (29)	Bristol-Myers Squibb	single drug:2.0%
	Pembrolizumab (29)	Merck Sharp Dohme(MSD)	single drug:0.4% Combination with pembrolizumab:2.0%
	Toripalimab	Shanghai Junshi Biosciences	0.7%(Package Insert)
	Sintilimab (30)	Innovent Biologics	1%
	Camrelizumab	Jiangsu Hengrui Pharmaceuticals	0.6%(Package Insert)
	Tislelizumab	BeOne Medicines	1.8%(Package Insert)
	Penpulimab	Kangfang Biology	0.6%(Package Insert)
	Zimberelimab	Gloria Biosciences	2.7%(Package Insert)
	Serplulimab	Shanghai Henlius Biopharmaceutical	0.8%(Package Insert)
	Pucotenlimab	Lepu Biopharma	2.7%(Package Insert)
PD-L1 inhibitor	Durvalumab	AstraZeneca	<0.1%(Package Insert)
	Atezolizumab (29)	Roche	1.4%
	Envafolimab	Alphamab Oncology	2.1%(Package Insert)
	Sugemalimab	CStone Pharmaceuticals	1.3%(Package Insert)
	Adebrelimab	Jiangsu Hengrui Pharmaceuticals	0.3%(Package Insert)
	Socazolimab (31)	Lees Pharmaceutical Holdings Limited	0%
	Benmelstobart	Chia Tai Tianqing Pharmaceutical Group	Unknown
	Avelumab	Merck/Pfizer	0.1%
CTLA-4 inhibitor	Ipilimumab (29)	Bristol-Myers Squibb	<0.1%(Package Insert) Combination with pembrolizumab:2.0%
PD-1/CTLA-4 Bispecific antibody	Cadonilimab	Kangfang Biology	0.9%(Package Insert)
PD-1/VEGF Bispecific antibody	Ivonescimab	Kangfang Biology	Unknown

The presence of islet-related antibodies is associated with a high prevalence of DKA. In a retrospective analysis of 172 cases of immunotherapy-induced diabetes, Liu (15) found that patients positive for glutamic acid decarboxylase antibody (GADA) developed ICI-induced diabetes earlier, with a median time of 7 weeks versus 16 weeks for GADA-negative patients (*P* < 0.001), and experienced a higher incidence of DKA (82.8% vs 62.1%, *P* = 0.006). Similar findings were reported by Byun (32). Wang (33) recently observed that GADA positivity may correlate with acute exacerbations and disease progression in some Japanese patients with ICI-T1DM. Consequently, measuring GADA prior to immunotherapy is advisable, with positive cases regarded as high-risk and monitored regularly following treatment initiation.

Notably, the patient received cadonilimab, a bispecific antibody targeting both PD-1 and CTLA-4. Dual immune checkpoint blockade is known to carry a higher risk of immune-related adverse events and more severe immune toxicity compared with anti-PD-1 or anti-PD-L1 monotherapy. This heightened immune activation likely accelerated pancreatic β-cell destruction, explaining the acute, life-threatening diabetic ketoacidosis observed here. Consequently, this case underscores the distinct endocrine toxicity risk posed by bispecific dual-target immune checkpoint inhibitors, warranting increased clinical vigilance. In addition, the patient received combination therapy with anlotinib before DKA onset. Anlotinib is a multi-target anti-angiogenic agent that may interfere with glucose metabolism and insulin sensitivity. When combined with Cadonilimab-mediated dual immune checkpoint blockade, anlotinib may exert a synergistic effect to exacerbate immune-mediated pancreatic β-cell destruction, thereby increasing the risk of severe ICI-DM and DKA. This potential combination-related risk warrants clinical attention.

### 3.3 Re-challenging with immune checkpoint inhibitors

#### 3.3.1 The necessity and safety of immune re-challenge

The decision to re-challenge a patient with immune checkpoint inhibitors (ICIs) following an immune-related adverse event (irAE) first requires determining whether alternative treatments with a greater potential benefit exist. While effective, ICIs are not the only option, targeted therapy, chemotherapy, and local interventions for oligoprogressive disease have proven efficacy in second-line and other settings, and their respective response rates and toxicities warrant careful consideration (34).

A subsequent consideration is the safety profile of ICI re-administration. Current evidence regarding the safety of continuing ICIs after endocrine irAEs remains limited. Zhao et al. (35) investigated the safety and efficacy of ICI re-challenge following an irAE, reporting combined incidence rates for all-grade and high-grade irAEs of 34.2% and 11.7%, respectively. Re-challenge was associated with a significantly higher incidence of all-grade irAEs compared to initial treatment (OR = 3.81; 95%*CI* = 2.15-6.74; *P* < 0.0001), though the incidence of high-grade irAEs was similar (*P*>0.05). Gastrointestinal irAEs and the initial irAE were linked to a higher recurrence rate of high-grade irAEs (*P* < 0.05), whereas an initial PD-1/PD-L1 regimen correlated with a lower recurrence rate (*P* < 0.05). Re-challenge with PD-1/PD-L1 inhibitors was also associated with a lower recurrence rate for all-grade irAEs (*P* < 0.05). The combined objective and disease control rates after re-challenge were 43.1% and 71.9%, respectively, showing no significant difference from initial ICI therapy (*P*>0.05). In a separate observational, cross-sectional pharmacovigilance cohort study, Charles et al. (36) analyzed 24,079 irAE cases and found that among 6,123 re-challenged patients, the recurrence rate of the original adverse event was 28.8%. Colitis, hepatitis, and pneumonitis had higher recurrence rates than other irAEs. Notably, over half of the patients whose initial irAE was diabetes opted for re-challenge, and among 13 cases with complete outcome data, none experienced a recurrence of diabetes.

Data on ICI re-challenge in gynecological cancers are particularly scarce. Kim et al (37) reported that a second course of ICI treatment after initial discontinuation yielded a disease control rate of 40%, with a median progression-free survival of 1.8 months (range 0.4-10.4) and a median overall survival of 21.3 months (range 10.1-52.7), patients who had discontinued due to an adverse event did not experience its recurrence during re-treatment. In summary, re-challenging with ICIs shows a manageable safety profile and retained efficacy, representing a viable treatment option for select patients who previously discontinued therapy due to an irAE.

#### 3.3.2 Recommendations for the management of ICI-T1DM

The NCCN and CSCO Guidelines for Management of Immune Checkpoint Inhibitor-Related Toxicities recommend that patients with positive DKA tests suspend ICI treatment, be hospitalized for consultation with an endocrinologist, and follow institutional DKA management protocols, including insulin therapy guided by the inpatient team or endocrinologist (4, 6). The 2020 Expert Consensus on irAE of the endocrine system induced by ICI states that insulin therapy should be initiated as early as possible for ICI-T1DM, while corticosteroids are not recommended; the primary treatment aims are to correct dehydration, hyperglycemia, and electrolyte disturbances (38). The development of ICI-T1DM is not a contraindication for continuing ICI therapy, and patients can proceed with immunotherapy while on insulin, for those with more severe disease (grade 2 and above), immunotherapy may resume once DKA has been corrected and qlucose level has stabilized. While ICI rechallenge is not absolutely contraindicated following the resolution of DKA and hyperglycemia in ICI-T1DM, cadonilimab was permanently discontinued for this patient after comprehensive clinical assessment. The decision was based on the patient’s advanced age of 81 years and relatively poor physical status. Concurrent upper gastrointestinal bleeding during the DKA episode further elevated her clinical instability and hemorrhagic risk. Our oncology team concluded that the risks of rechallenge in this context outweighed its potential therapeutic benefit. Consequently, treatment was permanently withdrawn to prioritize patient safety, aligning with the management principle for severe irAEs in vulnerable individuals.

#### 3.3.3 Strategies for immune re-challenge

Re-challenge strategies for immune checkpoint inhibitors (ICIs) involve administering a single ICI, dual ICIs, or ICIs combined with other anticancer agents. Currently, clinical data on ICI re-challenge remain limited. Guidelines from the National Comprehensive Cancer Network, the European Society for Medical Oncology, and the Society for Immunotherapy of Cancer support immunotherapy re-challenge for melanoma (39–41). Whether the initial regimen or an alternative ICI should be used in re-challenge trials is not yet established. Theoretically, employing a different ICI for re-challenge alongside anti-inflammatory strategies could preserve efficacy while reducing immune-related adverse events (irAEs). Combining ICI re-challenge with anti-inflammatory therapy may therefore enhance both safety and treatment outcomes; for example, concurrent blockade of interleukin-6 (IL-6) or tumor necrosis factor (TNF) with ICI therapy has been shown to mitigate irAEs, as demonstrated with the anti-IL-6 antibody tocilizumab, which can maintain antitumor activity while limiting toxicity (42–44). A lung cancer patient who developed diabetic ketoacidosis following camrelizumab treatment and was subsequently re-challenged with sintilimab, an agent associated with a lower incidence of hyperglycemia. After switching to sintilimab, hyperglycemia did not recur (45). This should not be interpreted as recovery of β-cell function, but is more likely due to the different immunogenicity of sintilimab compared with other ICIs. Different immune checkpoint inhibitors exhibit distinct safety profiles, and the risk of immune-related diabetes varies among different agents. Thus, selecting agents with a more favorable metabolic profile during re-challenge represents another viable therapeutic approach.

#### 3.3.4 Factors affecting the efficacy of immune re-challenge therapy

The factors influencing the efficacy of re-challenge therapy remain undefined. For ICI therapy, prior clinical benefit is widely considered the primary determinant for drug selection in a re-challenge, followed by performance status, PD-L1 expression level, and patient age (46, 47). Univariate logistic regression identified treatment discontinuation due to adverse events or clinical decision, absence of systemic therapy between ICI courses, a short inter-treatment interval, a low ECOG performance status, oligometastatic disease, and lack of brain metastases as positive factors for prolonged progression-free survival (PFS), whereas bone metastasis was a risk factor. Subsequent multivariate analysis confirmed that ECOG performance status influenced PFS during reactivation, while the interval between ICI treatments did not (48). As previously noted, a longer interval before ICI re-challenge was associated with a higher recurrence rate of high-grade irAEs (*P* < 0.05) (35). Han et al. proposed that immune memory from the initial treatment may persist after its cessation; as this response wanes, the established tumor dormancy can be disrupted, leading to loss of tumor growth control (47). Re-establishing control may require considerable effort, leading to their recommendation for re-challenge within three months. Further research is necessary to define the optimal patient population and treatment regimen for ICI re-challenge.

### 3.4 Clinical recommendations

The development of ICI-T1DM can be life-threatening, therefor routine monitoring of blood glucose, HbA1c, C-peptide, and islet auto-antibodies is recommended before and during treatment with Cadonilimab or other immune checkpoint inhibitors. Early recognition of the classic HbA1c paradox (severe hyperglycemia with only mildly elevated HbA1c) is critical for the early diagnosis of ICI-induced type 1 diabetes. Prompt insulin intervention is the first-line treatment for ICI-DM, and corticosteroids are not recommended because they may exacerbate hyperglycemia and diabetic ketoacidosis (4, 49). Clinical pharmacists can play an important role in glucose monitoring, patient education, medication adherence, and early identification of glycemic disorders in cancer patients receiving ICIs, leading to better health related outcomes and improved patient’s quality (49). For patients receiving dual-target ICIs (such as Cadonilimab), the risk of ICI-T1DM and DKA is higher, more intensive monitoring is warranted.

## 4 Conclusion

This case report details a patient who developed diabetic ketoacidosis (DKA) following four cycles of Cadonilimab therapy. Immunotherapy was discontinued, and the patient’s blood glucose was successfully controlled with standard fluid replacement and glycemic management. Cadonilimab treatment was not resumed. Immune checkpoint inhibitor-induced type 1 diabetes mellitus (ICI-T1DM) is a rare adverse event, necessitating close blood glucose monitoring in patients receiving ICI therapy. Most reported cases are linked to PD-(L)1 inhibitors, used alone or in combination, though the precise pathogenic mechanisms are not fully elucidated. At presentation, patients typically exhibit severe hyperglycemia and markedly low C-peptide levels. Many present with DKA, some have detectable islet autoantibodies, and others may show pancreatic atrophy or elevated lipase. Although immunotherapy may be cautiously continued if glycemic control is achieved, further clinical studies are required to define suitable candidates and protocols for re-challenge. Consequently, we recommend monitoring pertinent endocrine parameters-including blood glucose, glycated hemoglobin, fasting insulin, fasting C-peptide, diabetes-associated antibodies, thyroid function, and pituitary function-before and during ICI treatment. Maintaining vigilance for immune-related adverse events and initiating prompt intervention are crucial for ensuring patient safety.

## Data Availability

The original contributions presented in the study are included in the article/supplementary material. Further inquiries can be directed to the corresponding author.
